# High-throughput, multi-parametric, and correlative fluorescence lifetime imaging

**DOI:** 10.1088/2050-6120/ab7364

**Published:** 2020-02-19

**Authors:** Chetan Poudel, Ioanna Mela, Clemens F Kaminski

**Affiliations:** mab73641 Department of Chemical Engineering and Biotechnology, Philippa Fawcett Drive, University of Cambridge, Cambridge CB3 0AS, United Kingdom; cfk23@cam.ac.uk

**Keywords:** fluorescence lifetime, acquisition time, SPAD array, high throughput, FLIM, multi-parametric imaging, correlative FLIM

## Abstract

In this review, we discuss methods and advancements in fluorescence lifetime imaging microscopy that permit measurements to be performed at faster speed and higher resolution than previously possible. We review fast single-photon timing technologies and the use of parallelized detection schemes to enable high-throughput and high content imaging applications. We appraise different technological implementations of fluorescence lifetime imaging, primarily in the time-domain. We also review combinations of fluorescence lifetime with other imaging modalities to capture multi-dimensional and correlative information from a single sample. Throughout the review, we focus on applications in biomedical research. We conclude with a critical outlook on current challenges and future opportunities in this rapidly developing field.

## 1. Introduction

The fluorescence lifetime *τ* represents the average time a fluorescent molecule spends in its excited state before decaying to the ground state. *τ* depends on the molecule’s conformational state and immediate environment, and the decay kinetics can follow a single-exponential or multi-exponential decay function. For biological reporter molecules *τ* is typically of the order of nanoseconds. The fluorescence lifetime can be used as a quantitative sensor for various biophysical and chemical parameters in the fluorophore’s micro-environment [1, 2] such as pH, viscosity, temperature, ion concentrations and chemical reaction kinetics. It can also provide information on protein conformations and interactions through changes in *τ* via Förster Resonance Energy Transfer (FRET) [1, 3–5]. Fluorescence Lifetime Imaging Microscopy (FLIM) exploits the merging of lifetime measurements with imaging, making it a powerful quantitative imaging technique. A crucial advantage of FLIM is that the image contrast is based on measurement of the fluorescence lifetime, which is largely independent of the signal brightness and fluorophore concentration. This allows FLIM to circumvent many of the quantification challenges inherent in conventional intensity-based imaging [2].

The potential of FLIM is ever-increasing, particularly for applications in high-throughput, high-content drug screening, in clinical diagnostics, and for the study of biochemical reactions. However, the slow acquisition speed of FLIM, typically of the order of minutes per image, has remained one of its primary limitations. This has prevented the use of FLIM in observing dynamic phenomena, e.g. small moving micro-organisms, motion of cellular organelles, trafficking of vesicles and proteins, calcium and other ion dynamics, and other fast biophysical processes. To achieve the full potential of FLIM, fast image acquisition speed is required, either to permit increased temporal sampling of dynamic events or for high-throughput screening applications. While excellent books and reviews on FLIM applications and technical implementations already exist in the literature [1–3, 6, 7], a comprehensive review focusing on advances to enhance acquisition speed and image-throughput of FLIM has not been published. In this article, we review recent developments and the current state-of-the-art in this context. We will discuss in detail the inherent limitations of photon timing, advancements and challenges brought by massively-parallelised detection technologies, and efforts to develop real-time FLIM by incorporating data analysis methods with the physical image acquisition process. Finally, we briefly explore the use of FLIM for the acquisition of large multi-parametric datasets and conclude with an outlook to the future of the field.

## 2. High-throughput FLIM

Imaging methods based simply on capturing intensity (without the capture of time-resolved information) are able to produce reliable results with tens of photons per image pixel. On the other hand, time-resolved imaging techniques like FLIM typically require at least hundreds of photons [8] per pixel, even in ideal cases, for accurate determination of the fluorescence lifetime of samples. For this reason, obtaining high frame rates in FLIM is much more demanding than in intensity-based imaging. FLIM acquisition times per image typically range from tens of seconds to minutes, severely limiting the throughput of the technique. Various implementations of FLIM have been developed to increase the acquisition speed. The choice of technical implementation can impact the usable photon flux, speed, measurement accuracy, lifetime resolution, optical sectioning capability, and the compatibility with biological samples. In this article, we will focus on time-domain FLIM implementations but we also include a brief discussion of frequency-domain FLIM. Time-domain FLIM can be divided primarily into two broad categories: time-correlated single photon counting and time-gated FLIM.

### 2.1. Time-correlated single photon counting

**Figure 1.**
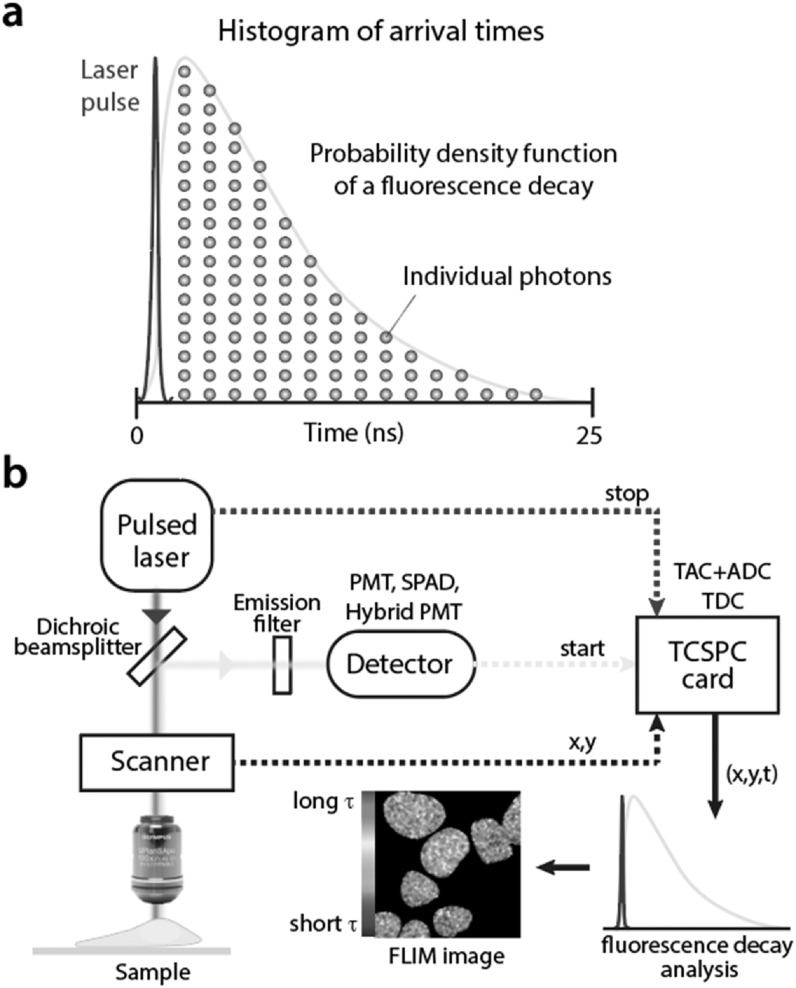
(a) The TCSPC principle: the iterative collection and sorting of photon arrival times into a histogram results in a probability density function of the fluorescence decay. (b) A schematic of a single-channel TCSPC-based scanning microscope. It employs a pulsed laser for excitation, which is focused onto a spot and scanned across the sample. Emission is collected with a single point detector (such as a PMT, SPAD or hybrid PMT). A TCSPC card receives electronic start and stop signals from the detector and the laser to time the arrival of photons. From the scanner, the TCSPC card also receives information about the position of the laser spot. This is important in assigning the photon arrivals to the correct image pixel. Once the acquisition is complete, the fluorescence decay information is analysed for each pixel and a FLIM image is generated.

Time-Correlated Single Photon Counting (TCSPC) is the most widely used FLIM implementation. In TCSPC, light pulses are used to excite a fluorescent sample. During the decay of fluorescence from excited state back to its ground state, photons are emitted from the sample and the arrival of these photons to the detector are individually timed in reference to the excitation light pulse. Photons are timed iteratively over a large number of pulses [9] until a sufficient number of photons are collected per pixel (usually more than 100 photons/pixel). For each pixel, the photon arrival times are sorted into a histogram which represents the Probability Density Function (PDF) of the fluorescence decay (see figure 1(a)). Since the histogram contains a large number of time-bins, the complete shape of the PDF is captured. Having the complete PDF information allows for an accurate estimation of the underlying fluorescence decay constants (fluorescence lifetimes) and their relative contributions to the decay. This is possible even for signals with multiple decay components, provided that a sufficient number of photons are collected. Other advantages of TCSPC include high signal-to-noise ratio and and high photon efficiency [6]. The latter arises from the fact that information from almost all photons reaching the detector is recorded without loss. This makes TCSPC-FLIM especially useful for the imaging of dim fluorophores, e.g. genetically encoded fluorophores in living biological samples [10].

TCSPC-FLIM is conventionally implemented using pulsed lasers, a point-scanning confocal microscope (which also enables optical sectioning capability), a point-detector and photon timing electronics (see figure 1(b)). There are a variety of detectors with single photon sensitivity that have been used in FLIM setups: photomultiplier tubes (PMTs), single photon avalanche diodes [11] (SPAD), or hybrid PMTs (which use a combination of PMT and SPAD technology). The timing electronics usually are a combination of Time-to-Amplitude Conversion (TAC) and Analog-to-Digital Conversion (ADC) hardware. In recent FLIM developments, Time-to-Digital Conversion (TDC) hardware have also been reported. A range of these electronic modules are available commercially with large on-board memories and some can support multiple timing channels. TCSPC provides excellent temporal resolution. The temporal resolution of the microscope refers to the smallest lifetimes that can be measured and for TCSPC, it is in the order of few tens of picoseconds. Temporal resolution is affected by the excitation pulse width, the response time of the detector and jitter (noise) in the electronics used. In general, FLIM implementations that use detectors with multiple units or 2D detectors (such as cameras) have longer instrument response times and therefore lower temporal resolution.

Photon detection and timing in TCSPC can be achieved through four main hardware architectures, each with distinct advantages and limitations in acquisition throughput, discussed below.

#### 2.1.1. Classical TCSPC: single channel detection and timing

**Figure 2.**
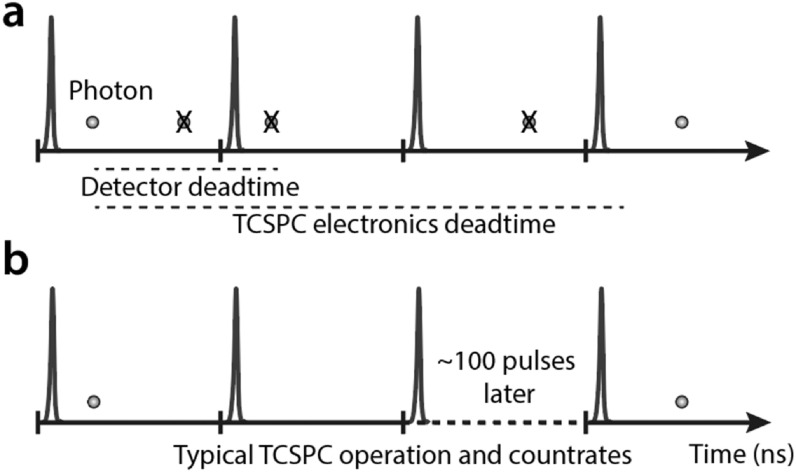
(a) When photon collection rates are high in TCSPC, the instrument dead times result in loss of information (X) of photons arriving late. This skews the probability density function towards shorter lifetime values and also results in loss of photon efficiency. (b) Typical TCSPC systems therefore operate at low laser powers and low photon count rates (∼1 photon/100 excitation pulses) to avoid photon pileup artefacts. This results in acquisition times of the order of minutes per image. Figure adapted from Wahl *et al* [13].

Classical TCSPC relies on a single detector in conjunction with electronics that can only time the arrival of a single photon reaching the detector at any given time. The combination of point-scanning and individually timing a large number of photons in a single timing channel makes image acquisition very slow, typically a few minutes per image. The most important limiting factor in acquisition speed is the dead time, in other words, the time required for the instrument to reset its timing circuitry after the detection of a photon. During the dead time, the instrument is insensitive to the detection of photons. The overall dead time includes those of the detector (∼10–25 ns) and the TCSPC electronics (∼25–200 ns) and can span multiple cycles of excitation. During this time, any photon arriving in the instrument is discarded (see figure 2(a)) and time-of-arrival information is lost, reducing the photon efficiency and placing an upper limit on the usable photon flux to 1 photon per dead time interval. Loss in photon efficiency may be considered acceptable in applications with bright samples but there is another important reason to keep the photon collection rate below this limit. In the case of samples with high photon counts where multiple photons can arrive at the detector after an excitation pulse, only the early-arriving photons are recorded and the late-arrivals are discarded. This skews the fluorescence decay PDFs towards shorter lifetimes. This distortion is referred to as photon pileup [12] and must be avoided in photon timing applications. This can be achieved by lowering the photon collection rate. Solutions to correct for photon pileup after acquisition do exist but are difficult to implement accurately. Therefore, even though modern detectors can provide very high detection rates (up to 40 MHz), most photon timing applications use very low collection rates to avoid photon pileup (collection rates of the order of 1 photon per 100 excitation cycles, which is ∼0.4 MHz for a 40 MHz pulsed laser, see figure 2(b)). The low photon collection rates increase the time required for each measurement, making single channel TCSPC-FLIM generally unsuitable for imaging dynamic samples or for high-throughput assays. Fast events like vesicle trafficking or movement of small organisms, cells, and organelles that occur over a few seconds introduce blurring artefacts and loss of spatial resolution in classical TCSPC and cannot be reliably monitored.

**Figure 3.**
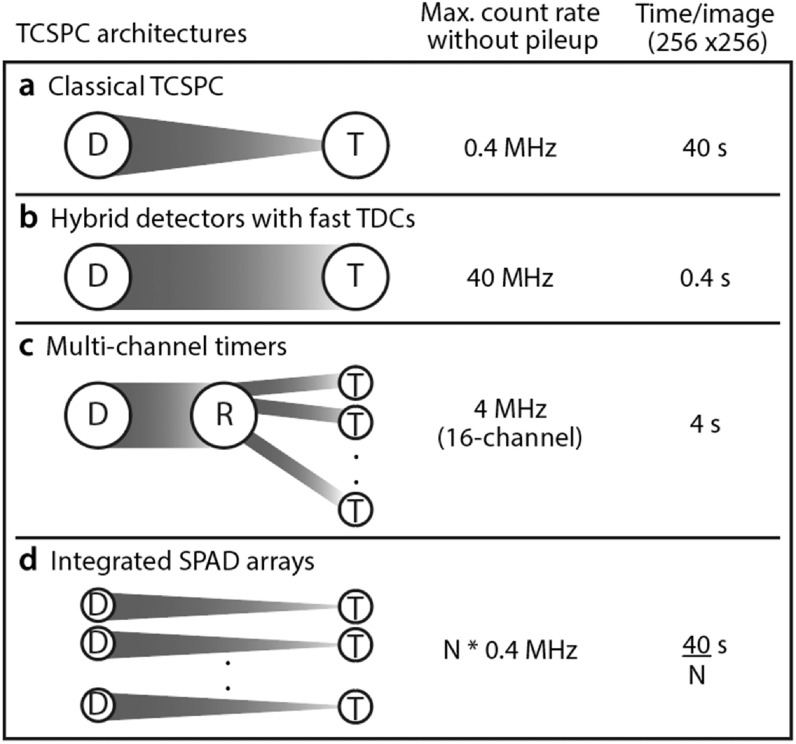
Four different sensor architectures for TCSPC FLIM. (a) Classical TCSPC with one detector (D) and one timer (T), where the rate of photon timing is the bottleneck in acquisition. To avoid pileup effects, we assume a safe photon count rate of 1% of laser pulses (∼0.4 MHz photon counts for 40 MHz laser repetition rate) for classical TCSPC. (b) Fast TDCs with negligible dead times remove the bottleneck in photon timing so that high count rates (∼40 MHz) without pileup artefacts are possible. (c) Multi-channel architectures use a router (R) to send detected photons into multiple timing channels, which is an effective way to parallelise and increase the limited timing rate (∼4 MHz total offered commercially for 16-channel TCSPC modules). (d) SPAD arrays are scalable versions of classical TCSPC systems with a large number of integrated timing elements. When coupled with efficient light collection systems, they offer scalable enhancements in recording speed. The time/image in the figure is estimated assuming a 256 × 256 image with 250 photons in each pixel and high photon emission rates from the sample. In practice, dimmer samples and non-ideal light collection will increase the acquisition time significantly. Figure adapted from Arlt *et al* [12].

There have been some noteworthy attempts to incorporate TCSPC systems into automated plate-readers or flow cytometry for high-throughout screening or for dynamic imaging [14–16]. However, these attempts either sacrifice spatial information altogether to extract a single lifetime measurement, or they sacrifice accuracy and introduce bias by using high count rates and ignoring photon pileup. Performing fast FLIM without such significant sacrifices necessitates the use of faster timing systems or parallelised detection schemes (see figure 3).

#### 2.1.2. Single-channel detection with hybrid detectors and fast timing electronics

It is immediately obvious that the photon throughput and pileup problem in single-channel TCSPC can be improved by reducing the dead times of the detector and timing electronics. Hybrid PMTs use a combination of PMT and SPAD technologies and feature lower dead times (<1 ns) compared to either PMTs or SPADs (tens of ns). Hybrid PMTs have therefore become the favored detectors for photon timing [17, 18]. Similarly, there have been major advances in the technology of timing electronics and fast TACs and TDCs have been developed, which feature very short dead times (<1 ns), and are now commercially available. Combining these faster electronics with hybrid PMTs effectively removes the timing bottleneck in TCSPC (see figure 1(b)), allowing for very high photon timing rates, upto tens of MHz. This has formed the basis of fast commercial FLIM systems such as rapidFLIM [19].

In theory, acquisition speed can be increased by two orders of magnitude over classical TCSPC with only slight losses in accuracy. There is still room for improvement in shortening the dead times of FLIM hardware to improve accuracy at high photon count rates. On the other hand, measurements of very slow decays, e.g. in luminescence and phosphorescence can also benefit greatly from improved capabilities of detection electronics, permitting multiple photons to be timed for a single laser pulse [13]. However, these improvements come with electronic jitter leading to sacrifices in temporal resolution (∼250 ps versus ∼20 ps for classical TCSPC). This may limit applications requiring measurements of very short fluorescence lifetimes. Most biochemical imaging assays can afford this loss of temporal resolution as typical fluorescence lifetimes are in the order of nanoseconds.

#### 2.1.3. Multi-channel timing with single or multiple detectors

The speed limiting step in single-channel TCSPC is the slow rate of photon timing. One way of solving this problem is the parallelisation of timing by routing the detected photons over multiple timing channels (usually 8 or 16, see figure 3(c)). In this way, multi-channel TCSPC modules offer an alternative means to achieve high photon throughput by decreasing the chances of photon pileup on any single channel. For instance, a commercial 16-channel system offers an order of magnitude improvement in photon throughput (∼4 MHz) over classical TCSPC (∼0.4 MHz). The temporal resolution is worse (∼200 ps) but this does not pose a significant limitation for many practical FLIM applications.

For imaging very bright samples, a separate detection unit for each timing channel can also be implemented. Such PMT arrays containing 16 independent detector-timer architectures have previously been used for capturing many emission beams created by multifocal excitation [20, 21]. Dispersing a single emission beam with a prism onto the PMT array can also enable wavelength-resolved FLIM capabilities. However, detector arrays also have limitations. They can suffer from optical cross-talk between individual units, which needs to be characterised and corrected. Scaling up from 8 or 16 TCSPC channels using this technology can also be prohibitively expensive. Viable alternatives in parallel detection emerged with the introduction of large arrays of TCSPC-enabled SPAD pixels and chips, discussed next.

#### 2.1.4. SPAD arrays

In the last decade, prototypes of SPAD arrays fabricated on single monolithic chips have been reported. Examples include linear arrays [22–27] or rectangular arrays [28–32] with tens or hundreds of SPAD units. The arrays feature TCSPC signal processing capabilities for each SPAD unit/pixel [27, 33] or for the entire array chip [34, 35]. Arrays with in-pixel TCSPC have, in each pixel, dedicated electronics assembled right next to the detector’s ‘active’ area where photons are detected. Each such integrated pixel suffers from the same limitations as single-channel TCSPC but the array as a whole with many independent units offers a highly scalable parallelisation of FLIM acquisition (see figure 3(d)). One limitation of SPAD arrays is their low fill factor. Fill factor is the proportion of the total detector area that is ‘active’ or capable of detecting photons. SPADs feature small active areas (tens of micrometers in diameter), and exact alignment of light onto the active area is a critical requirement. SPAD arrays also suffer from higher dark count rates than typical PMTs. Dark counts arise from electrons in the detector spontaneously triggering a count without the arrival of a photon. The internal architecture of SPAD arrays and overheating makes them more susceptible to producing dark counts. Such electrical noise degrades the signal-to-noise ratio of the images but can be mitigated by implementing detector cooling systems. In SPAD arrays with large number of detector elements, the data transfer rate from many parallel TCSPC circuits to the computer can itself be a major limitation. As an example, a paper [31] reported that data transfer limitations restricted the data acquisition in a 32 × 32 SPAD array to only using 64 detectors. These limitations will be critical in widefield TCSPC implementations but will hopefully be eliminated in the future with faster bus interfaces.

SPAD arrays can be implemented with both point-excitation and in widefield mode (discussed later). When using point-excitation, data can can collected with SPAD arrays in three primary modes. All of these modes allow fast, high-throughput imaging via massively parallelised acquisition:

a. Scanning a single-spot at high excitation power and distributing the emission across all array units [29]
b. Scanning of multiple excitation spots, and mapping emission spots onto the array [24, 31]
c. Using prisms before the array to disperse the emission beam for spectral-FLIM capabilities [27]

In the first mode, photon flux from a single high intensity laser spot is distributed over a large number of indpendent detector-timer units. This parallelisation reduces acquisition times by a linear factor which is proportional to the number of SPAD units in the array. Photons from all SPAD pixels can be pooled for a single high-speed measurement with an extended dynamic range [29]. This means that a larger range of signal intensities can be covered, from low background to very bright pixels in the same image, by distributing the signal into many detectors and raising the total upper limit of detectable photons. Simple optical schemes that homogeneously distribute the emission onto the array result in large loss of photons in the inactive areas. Therefore, this implementation is limited to bright and photostable samples that can afford photon losses.

**Figure 4.**
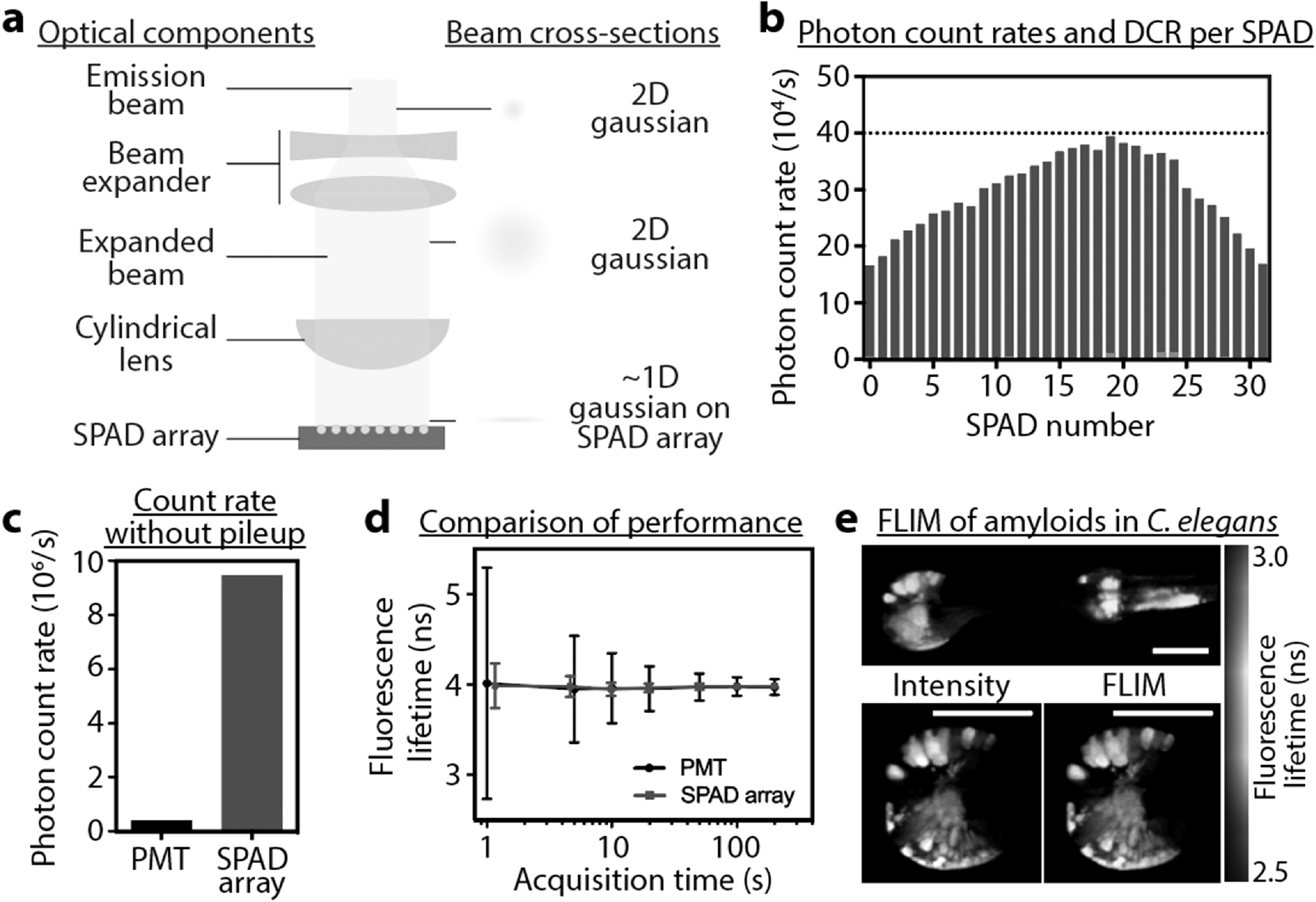
(a) A simplified optical setup of the emission arm between the microscope output and SPAD array is shown. A cylindrical lens shapes the emission beam to a 1D gaussian profile onto the 32 × 1 SPAD array detector. (b) Typical illumination profile for the SPAD array (obtained experimentally) shows a distorted 1D gaussian. Laser power was capped at a point where the SPAD unit with the highest count rate was still under 1% of 40 MHz laser repetition rate (dotted line) to reduce the effects of photon pileup. Dark count rate is plotted with red bars but is negligible in the figure for most SPAD units. (c) Photon count rates summed up for all 32 SPAD units is ∼23 times higher than the maximum achievable count rate of a single PMT (0.4 MHz), while ensuring both SPAD array and PMT stay under 1% photon pileup. (d) A direct comparison of measurement precision (variability) between the SPAD array and a standard PMT was performed by measuring the fluorescence lifetime of Rhodamine6G (4.08 ns). To achieve precision comparable to a standard PMT, the SPAD array took 1/20th of the time. (e) FLIM images of amyloid aggregation in C. elegans measured in 10 s using the SPAD array. Scale bar: 50 *μ*m.

In figure 4, we demonstrate this single-spot scanning mode using a linear 32 × 1 SPAD array, the technical details of which have been published [24, 25]. This implementation can, in theory, handle a photon flux up to 32 times higher than that of a single-channel TCSPC system without suffering from photon pileup. We used Rhodamine6G to demonstrate the principle and achieved around 20 times increase in acquisition speed, while maintaining similar lifetime accuracy and precision as single-channel TCSPC (see figure 4(d)). The non-uniform distribution of emission onto the array (see figure 4(b)) decreased the overall signal flux and thus reduced the potential gains in acquisition speed. Large photon losses due to low fill factor were compensated by illuminating the sample at more than 100 times higher laser powers. Moving towards a biologically relevant system, we investigated *C. elegans* models of neurodegeneration that overexpress proteins forming bright amyloid-like aggregates [36] (see figure 4(e)). We managed to reduce the acquisition times from the typical 2 min (with classical TCSPC) to only ∼10 s (with the SPAD array) to deliver a similar performance. Again, this was possible only because of the sample’s high brightness.

Unfortunately, imaging dim biological samples or capturing a time-lapse using this configuration is not possible due to severe photobleaching and phototoxicity caused by the high power laser illumination required. Photon efficiency is of utmost importance for live-cell applications. Photon losses can be mitigated by fabricating SPAD arrays with higher fill factors [26, 33, 37] or could be achieved by the use of microlens arrays [38]. Alternatively, more sophisticated illumination schemes can be used to improve photon efficiency and speed, discussed below.

The second mode of acqusition with SPAD arrays is technically more complex but a viable alternative for biological or live-cell FLIM. It involves the generation of multiple focal spots for excitation using microlens arrays [24] or spatial light modulators [31, 32, 39]. The sample is then scanned across the fixed excitation spots using a stage scanner. Dividing one high-intensity laser spot into many weaker multifocal spots is less phototoxic to cells but still allows maintaining high powers and photon throughput when summed over the entire sample plane. Emission from the spots is imaged onto the pixels of the SPAD array [31, 32]. Here, the imaging speed is proportional to the number of foci [24]. Simultaneous generation of spots in the axial rather than lateral dimension is also possible, which allows the collection of multiple *z*-planes in one lateral scan to create a 3D FLIM image [32]. The conjugate alignment of the illumination, sample and detection planes is critical in these multifocal scanning configurations to optimise the image resolution and detection efficiency [31]. This imaging mode has extended the range of biological FLIM applications to live-cell FRET and *in vivo* studies [31], thanks to the reduced phototoxicity. However, the complex intrumentation and alignment may limit its use to specialist imaging labs.

The third mode of image acquisition utilizes a prism before the SPAD array to disperse the beam and capture the entire wavelength spectrum of the emission simultaneously. Popleteeva *et al* used a 64 × 4 SPAD array to acquire such spectrally-resolved FLIM images [27]. Due to the large number of channels available, this system could provide higher spectral resolution than commercial state-of-the-art spectral-TCSPC detectors. Acquisition times for spectrally-resolved FLIM images were reduced from 360 s on a commercial TCSPC system to 8 s on this SPAD array. This SPAD array was also used to image samples that require sensitive detection of low-light signals, as in the case of tissue autofluorescence and FRET.

Overall, the rapid development of SPAD array technologies holds great promise for high-throughput FLIM, and for the development of even faster implementations like widefield TCSPC.

#### 2.1.5. Widefield TCSPC

All fast detection systems described so far are based on a scanning microscope. Therefore, the microscope scanner speed sets the upper limit for the frame rates possible. When higher frame rates (beyond ∼1 Hz) are necessary, a widefield system can be used to harness the simultaneous detection of all pixels. Widefield TCSPC retains a lot of the benefits of TCSPC (high temporal resolution and single photon sensitivity) but in general sacrifices optical sectioning capabilities. Widefield TCSPC requires position-sensitive single-photon detectors with picosecond time resolution to simultaneously capture the position and arrival time of photons [40, 41]. Photon-counting Micro-Channel Plates (MCPs) [42] and large SPAD arrays are both promising candiates for this application. Video rate FLIM (∼10 Hz) has been demonstrated [43] using SPAD arrays with high fill factor and image resolution. Villa *et al* have compared some relevant technical parameters for some of the existing SPAD imagers [44]. The number of SPAD pixels, fill factor and temporal resolution needs further improvement but even with incremental developments, we may expect commercial SPAD array cameras to provide widefield TCSPC capabilities in the near future. It is important to note in this context that while speed gain may be advantageous, this technique does suffer from the general problems of widefield imaging like out-of-focus fluorescence contamination and scattering in the optics. These problems can be mitigated by using illumination schemes that provide optical sectioning e.g. total internal reflection (TIR), super-critical angle fluorescence or light-sheet illumination. For more information on widefield TCSPC, we refer the reader to an excellent review [40] on methods and applications of widefield TCSPC.

### 2.2. Time-gated FLIM

**Figure 5.**
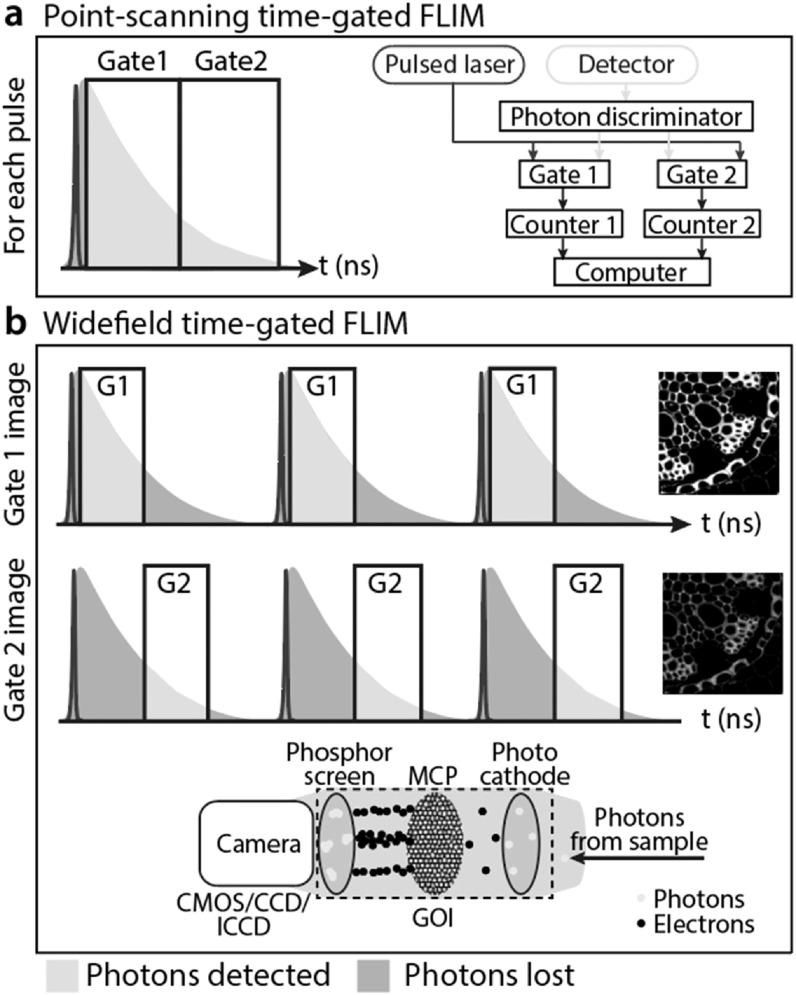
A schematic showing the operation of TGFLIM. (a) In point-scanning TGFLIM, multiple time gates (2 gates shown, left) are enabled sequentially for each excitation pulse. A photon discriminator (right) identifies photon signals arriving at the detector. These signals are split and routed into Gate1 or Gate2. The gates are enabled sequentially and each has a photon counter. (b) In widefield TGFLIM, one gate setting is enabled for each camera exposure. This results in a stack of images with decreasing intensities (one image per gate, see right). The operation of a GOI is shown in the bottom: emitted photons striking the photocathode create electrons. When the gate is enabled, the high gain voltage on the micro-channel plate (MCP) amplifies these electrons. These electrons strike a phosphor screen, which is then imaged by the camera. When the gate is turned off, the MCP voltage is zero and electrons are not amplified. The camera is still recording at this time but mostly sees a dark background until the gate turns on again at the next laser pulse. After a full camera exposure (say 50 ms), the gate is shifted temporally to its next position and the process begins again.

Time-gated FLIM (TGFLIM) is a FLIM implementation where the fluorescence time decays are sampled in two or more time windows or gates. Each gate is a time interval in which signals are integrated before the gate is closed again. The ways to set these time gates or intervals in the detection system differ between point-scanning and widefield implementations and will be discussed in their individual subsections below. In both cases, time gates are usually a few nanoseconds wide and delayed by varying offsets relative to the excitation pulse (see figure 5). TGFLIM circumvents the speed restrictions inherent to TCSPC approaches by avoiding entirely the timing of individual photons. The acquisition speed of TGFLIM is therefore faster than TCSPC and can be boosted further by detecting photons in multiple gates simultaneously [6, 45] rather than sequentially [46].

#### 2.2.1. Point-scanning TGFLIM

Point-scanning TGFLIM [8, 9, 47, 48] detects emitted photons originating from a single illuminated spot that arrive at a single or multichannel detector [27]. The signal from the detector is split and routed into two or more photon counters based on the time gate the signal arrives in. Many gates can be sequentially enabled after each laser pulse. Single-photon sensitivity is still achievable in point-scanning mode and the detection efficiency can be close to 100%: virtually the whole decay is detected after every excitation pulse [8] (see figure 5(a)). Detecting the signals in all gates for each pulse makes the microscope impervious to laser intensity fluctuations or photobleaching. The number and the width of gates can be controlled and ideally the gates have sharp (sub-nanosecond) onset times so that signals can be routed without delay. The precision of lifetime measurements in TGFLIM depends on the speed that the gates can be opened/closed (usually sub-ns) and also on the timing jitter of the detector. The detector dead time can be a limiting factor but can easily be improved by using hybrid PMTs or gated SPAD arrays [27]. Dead times for the counting electronics are usually small (<0.5 ns), and all gates can be reset in time for the arrival of signals originating from the next laser pulse. This allows for extremely high count rates and fast acquisition times limited no more by electronics but only by the scanning speed of the microscope (typical acqusition times are less than one second for a 256 × 256 image with 250 photons in each pixel [9]). Capturing the complete shape of the PDF using multiple time bins (as is achieved in TCSPC) is not necessarily required to obtain a useful indicator for lifetime changes. Two gates are sufficient for an estimation of the lifetime of a single-exponential decay or the average lifetime of a multi-exponential decay, since the ratio of signals received in the two gates is sensitive to the shape of the fluorescence decay curve. However, in a multi-exponential decay, multiple combination of lifetimes and their relative contributions can result in the same average lifetime. Therefore, sampling the fluorescence decay using more than two time-gates is required for accurately characterizing all the decay parameters in a multiexponential decay. Gerritsen *et al* compared the measurement sensitivity for implementations involving 2, 4 and 8 gates [48]. Not surprisingly, the use of a greater number of gates results in significantly improved lifetime estimates [9, 47] but comes at the cost of reduced acquisition speed.

#### 2.2.2. Widefield TGFLIM

To achieve high frame rates not limited by point-scanning, TGFLIM can be combined with widefield excitation and gating of the detector [46, 49]. Unlike in point-scanning TGFLIM in which signals from detected photons are selectively routed to different gates, in widefield TGFLIM the detector is gated directly like a fast shutter to accept or reject photons. If the photons reach the detector assembly within the specified time window/gate, they are successfully relayed to the camera (usually through photon amplification). Photons arriving outside this time gate are rejected (as they do not get amplified). Usually, only one time gate is enabled per laser pulse. For each gate, an image is constructed by integrating the fluorescence signal received over many thousands of pulses. To sample the fluorescence decay, multiple sequential acquisitions over all the different gates are required (see figure 5(a)).

Modern detectors for widefield time-gating use a Gated Optical Intensifier (GOI) or High Rate Imager (HRI) [50] with specially designed photocathodes and micro-channel plates to amplify photons and boost the signal-to-noise ratio. These are placed in front of image sensors like charge-coupled devices (CCDs), intensified-CCDs or streak cameras (see figure 5(b)). The photon amplifying gain on the GOI can be modulated to be produced at GHz frequencies, permitting very short gates. GOIs offer large versatility in selecting the gate widths (e.g. tens of picoseconds to 1 millisecond) with sharp onset times (picoseconds). They also work with a large range of repetition rates (e.g. from single shot to 110 MHz) [51].

Only one gate per pulse can be enabled in widefield TGFLIM using single detectors because of the short time scales involved. The photon efficiency of the system decreases proportionally with the number of sequential acquisitions (or number of gates). To improve photon efficiency and speed, elegant but complex solutions have been devised to capture multiple time gates simultaneously. This involves splitting the emission beam into multiple beams and then delaying them with respect to each other optically, and finally focusing them separately onto the same GOI. Agronskaia *et al* demonstrated this principle first [49] and were able to capture extremely fast calcium flux dynamics in myocytes using FLIM at frame rates reaching 100 Hz [52]. The gating hardware can be driven to capture even higher frame rates (∼800 MHz possible) but the low amount of fluorescence signals emitted by samples at such short timeframes make it impractical for most applications. Elson *et al* implemented a similar idea and recorded four gated images simultaneously onto a segmented GOI via beam splitting and optical delaying. This enabled video-rate or single-shot FLIM [53]. Overall, TGFLIM has enabled studies of dynamic phenomena [52, 54], clinical diagnosis [55] or high throughput, high-content screening for drug discovery and interactomics [56–58]. The ability of the method to discriminate small changes in lifetime is robust enough for sensitive FLIM-FRET experiments [51, 54, 56–59], even at high speeds.

**Figure 6.**
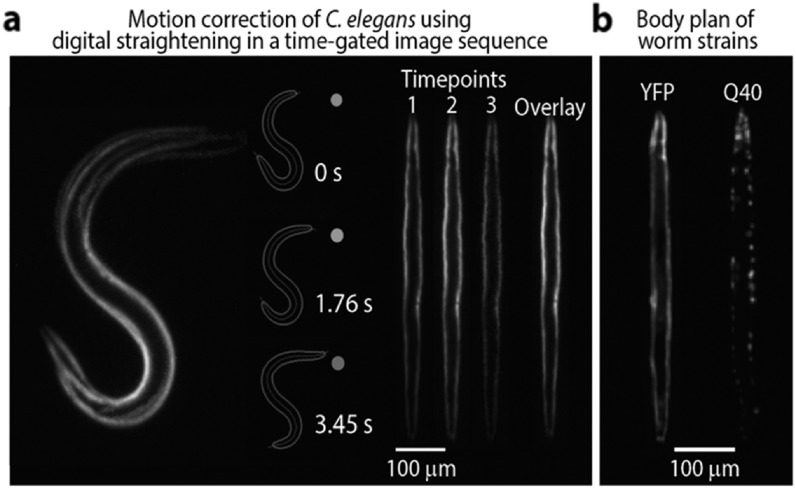
Correction of motion artefacts in FLIM post-acquisition. Images of C. elegans moving freely during sequential gating in a TGFLIM acquisition were digitally straightened. Laine *et al* [61] spatially corrected for 2D movements using skeletonisation, backbone straightening, and registration of all straightened worm images onto a common body template. Using motion correction also permitted visualising in the body of living C. elegans the spatial organisation of amyloid aggregates formed by protein mutants linked to Parkinson’s and Huntington’s diseases. Such digital correction requires samples with well-defined structures.

Fast widefield FLIM detection also comes with other benefits, for example allowing the correction of motion artefacts [60] for live-cell or *in vivo* endoscopic applications. Faster temporal sampling during acquisition avoids movement artefacts without the need for image processing [53]. However, even in cases where sample dynamics exceed imaging speed, motion artefacts can be accounted for in FLIM analysis in certain cases. Laine *et al* explored the digital straightening of sequential images of moving *C. elegans* to avoid motion artefacts in subsequent TGFLIM analysis [61] (see figure 6). The method exploits spatial correlations between subsequent images of known structures to account for sample motion, an approach not available to point-scanning methods.

However, widefield TGFLIM poses some inherent challenges. Widefield detection comes with a loss of spatial resolution, contrast and single photon sensitivity. In addition, photobleaching can introduce measurement bias towards shorter lifetimes in sequential gating by reducing the number of photons that would otherwise reach the detector at later time gates. Photobleaching therefore limits widefield TGFLIM to applications with bright and photostable fluorophores such as organic dyes. A permuted recording order of gates can partially suppress photobleaching artefacts [62]. The lack of a pinhole in widefield imaging can furthermore introduce artefacts from out-of-focus light, which contributes to the detected FLIM signal. One solution to counter both photobleaching and out-of-focus contamination is to use light sheet illumination which also provides higher image contrast and signal-to-noise ratio [63, 64]. TIRF [65] and spinning-disk (Nipkow) approaches [56, 57, 66] have also been used to gain an optical sectioning capability in some high-content FLIM microscopes. Acquisition speed may be somewhat compromised using a Nipkow disk (compared to standard widefield imaging) but it is still around 15 times faster than TCSPC-enabled confocal imaging for comparable lifetime accuracy [54].

Beside TCSPC and TGFLIM, a few other measurement schemes [67–69] have also been developed to increase acquisition speed in time-domain approaches but are not covered in this review.

### 2.3. Frequency domain FLIM

While this review is primarily focused on high-throughput time-domain FLIM methods, we also briefly mention noteworthy attempts in Frequency Domain FLIM (FD-FLIM). FD-FLIM is functionally analogous to time-gated approaches in time-domain FLIM and avoids the photon pileup problem of TCSPC. FD-FLIM uses modulated (sinusoidal, square-wave or pulsed) excitation waveforms in conjunction with phase-sensitive modulated detectors [70–72]. The lifetimes are determined from the phase shift or the demodulation of the fluorescence signal relative to the excitation waveform. Resolving complex decays requires sampling of the signal at different phases, which is typically performed sequentially. Using just three images acquired at different phases, high-speed widefield FD-FLIM operating at 8 Hz [73] and 5.5 Hz [72] have been demonstrated. The limitations caused by sequential phase sampling on the photon efficiency can be overcome by utilizing parallel retrieval of phase-dependent images in widefield mode [74]. This can make FD-FLIM comparable to TCSPC in terms of photon-efficiency and comparable to time-gated FLIM in terms of acquisition speed. FD-FLIM can be implemented not only in widefield [70–72, 75] but also in scanning [10, 76–78] mode. A highly photon-efficient and low-cost implementation has been shown for scanning microscopes, using a field-programmable gate array (FPGA) board for digital FD-FLIM [77].

The acquisition speed of FD-FLIM is comparable to fast time-domain implementations [79, 80]. FD-FLIM at ∼24 Hz using time-of-flight detection was demonstrated in 2004 [74]. Acquiring at high speed may require high excitation powers which introduce artefacts via photobleaching. A permuted recording order of phase images can allow for partial supression of such photobleaching-induced artefacts [62].

The first demonstration of using FLIM combined with automated microscopy for high-throughout screening was performed using FD-FLIM [81]. It was used to provide a basis for scalable, unsupervised screening platforms for the purpose of gathering multi-parametric and high content data at sub-cellular resolution. When paired with fast analysis techniques like phasor plots (see section 2.4), both imaging and analysis can be accomplished in real time.

Compact FD-FLIM cameras operating at high frame rates (tens of Hz) are now available commercially using modulated complementary metal-oxide-semiconductor (CMOS) sensors [82, 83] and CCD cameras [79, 84–86]. In some cases, data analysis has been integrated into the acquisition software, allowing for simple operation even for non-expert users.

A brief summary of the characteristics, strengths and limitations of various FLIM implementations is presented in table 1.

**Table 1.** Comparison of different FLIM implementations and their limitations and strengths.

FLIM implemen-tations	Single-channel TCSPC	TCSPC with SPAD arrays	Point-scanning TGFLIM	Widefield TGFLIM	FD-FLIM
Underlying technology	TACs or TDCs measuring arrival times of individual photons	Large number of one-to-one detector-timer connections	Fluorescence decay sampled at a point in several time-gates simultaneously	Fluorescence decay sampled for full field-of-view by sequential shifting of temporal gates	Modulated excitation and detection
Excitation	Pulsed femtosecond or picosecond lasers with MHz repetition rates				Modulated or pulsed lasers
Detection	PMT, SPAD, Hybrid PMT	SPAD arrays with in-pixel or on-chip circuitry for photon timing	MCP-PMTs	Gated ICCDs or sCMOS cameras with gated optical intensifiers	Modulated intensifiers or cameras
Microscopy platform	Point-scanning (with optical sectioning): confocal or two-photon microscopy; multi-spot scanning may be used with SPAD arrays			Widefield (optionally in conjugation with Nipkow disk for optical sectioning)	Point-scanning or widefield (or in conjugation with Nipkow disk) platforms
Photon efficiency	High	Limited by low fill factor of SPAD arrays	High: all gates measure in parallel	Moderate to low because of sequential time gating	Limited by requirement to measure at multiple phase steps
Image acquisition speed	Slow: typically minutes, unless fast TDCs are used	Depends on number of SPAD units: typically seconds but the technology is improving	Fast: typically seconds, limited mostly by scanning	Video rates demonstrated (10 s of Hz)	Video rates demonstrated (10 s of Hz)
Strengths	Highest temporal resolution and accuracy. Works best for static samples and low brightness samples.	Fast acquisition rates but need bright samples	Photon efficient	Fast due to parallel acquisition in all pixels.	Fast and cost-effective implementation.
Primary limitations	Speed is limiting for dynamic or high-throughput imaging.	Need for precise optical alignment; low fill factor of SPAD array; high DCR	Technically complex; speed limited by scanning systems.	Usually photon inefficient and prone to photobleaching artefacts	Multi-exponential decays are difficult to resolve.

### 2.4. Fast FLIM analysis and visualisation methods

In many high-throughput or clinical (diagnostic) applications, fast analysis and data visualisation are equally important as fast data acquisition. Research in FLIM data processing methods is thus an active field. In the time-domain where a complete decay curve is recorded as in TCSPC, traditional analysis methods like iterative fitting using Weighted Non-Linear Least Squares (WNLLS) algorithms are widely used, despite being slow and computationally expensive. Modern implementations, for example the open source package *FLIMfit* [87] uses parallel computation to batch-fit complex decay models using WNLLS on large FLIM datasets. This permits high-content analysis of multi-well plate data within tens of seconds on standard PCs [88]. However, this timescale is still restrictive for clinical applications like endoscopic FLIM where real time FLIM recording is a necessity.

Here, analytical methods such as Rapid Lifetime Determination (RLD) [89] can be used in combination with TGFLIM. RLD comes with only minor trade-offs in precision but orders of magnitude gains in processing speed. RLD uses the signals at different regions of the fluorescence decay curve to calculate fit parameters for both single [90] and multi-exponential decays [89], and works well in samples with known lifetimes. RLD reduces the lifetime calculation to a simple ratio for TGFLIM data captured using two gates [8] and offers real time processing capabilities [53].

In FD-FLIM, two lifetime estimates are obtained, respectively, from the demodulation and phase-lag between the reference measurement and the sample. The powerful phasor-plot analysis provides an efficient way to visualise these lifetimes, and offers a global overview of multiple lifetime components in each pixel without *a priori* information [91, 92]. This so called global analysis method has recently been integrated directly into acquisition software for real time visualisation of lifetime changes. The phasor-plot technique has also gained popularity in time-domain FLIM. It avoids problems inherent with exponential fitting, and provides for a natural representation of multi-component decays [93, 94]. Other time-efficient algorithms calculate moments of the lifetime distribution [95] which has enabled real time FD-FLIM to be performed [70, 96]. This method has also been adapted for analysing TCSPC data and reports greater accuracy compared to fitting procedures, in cases where short lifetimes need to be measured or the signal intensities are lower [97].

In cases where the lifetimes of multiple fluorescent species in the sample are invariant and only their relative concentrations are different in each image pixel, global analysis algorithms permit an immediate and intuitive interpretation of both time-domain [98, 99] and frequency domain [91, 100] data. Global analysis can significantly improve accuracy and precision of lifetime estimation compared to conventional pixel-by-pixel fitting for images with low signal-to-noise ratio. This includes almost all live-cell imaging and fast FLIM applications.

## 3. Multi-parametric and correlative FLIM

While faster, high-throughput FLIM approaches are critical in many applications, there is also need for high content and multi-parametric data from static samples or samples with slow dynamics. FLIM on its own enables diverse possibilities in biophysical characterisation but can be even more powerful when combined with other microscopy techniques. Advanced microscopes have thus been created [27, 101–103] to capture multi-dimensional datasets that contain not just the spatial profile and fluorescence lifetime of the sample but also the polarization anisotropy [104] and absorption-emission spectra *(x, y, z, τ, r, λ*_*ex*_*, λ*_*em*_) for each pixel.

Polarisation-resolved FLIM can be powerful when investigating the structural orientation, rotational dynamics, and functional aspects of proteins and protein complexes [105, 106], which may not be accomplished using FLIM alone [107–109] or may require techniques not compatible with living cells. Similarly, capturing FLIM at different absorption-emission spectra proves useful in the characterisation of autofluorescence of tissues, which is altered in certain diseased states [110]. Such multi-parametric imaging benefits from using wavelength-flexible excitation sources like a supercontinuuum [102, 110–113], which provides pulsed light in a range of excitation wavelengths. Capturing large multi-dimensional datasets can require long acquisition times in the order of tens of minutes. Yet, recent advances in microscopy instrumentation and software have enabled parallel detection of the full set of fluorescence properties in just one or two minutes, maximizing the biochemical resolving power of fluorescence microscopy [114]. The analytical tools to handle and visualise these complex datasets have been developed in unison. This combined approach of gathering, analysing and exploring the full optical properties of a sample enables detailed biochemical studies, e.g. of multiplexed signaling pathways, functional aspects of proteins at the molecular scale, or exploring properties of tissue biopsies in clinical diagnosis. When probing tissues, FLIM can be combined with multi-photon excitation enabling the label-free study of metabolic dysfunction and disease from tissue autofluorescence [115–118]. Multiphoton imaging also increases the imaging depth [119–121] for *in vivo* applications.

Some modalities integrate fluorescence recovery after photobleaching (FRAP) measurements simultaneously with anisotropy and lifetime in a single experiment [109], offering information about mobility, molecular conformation and the chemical environment of molecules, receptors or protein clusters. As an example, the characterisation of amyloid protein aggregates by FLIM [122] was combined with FRAP experiments to distinguish protein clusters that are mobile or immobile.

**Figure 7.**
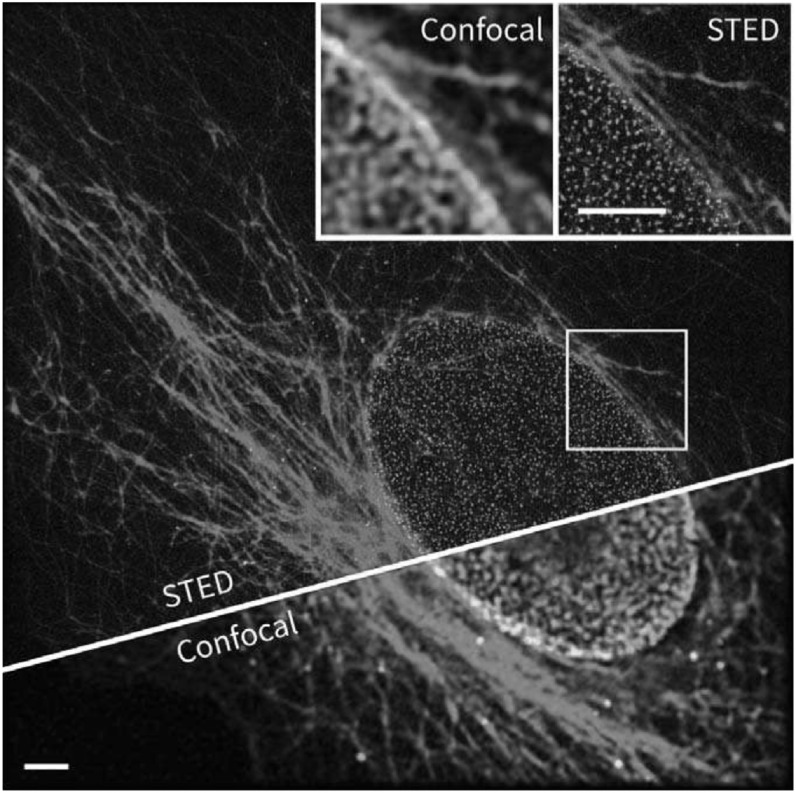
STED and confocal images of *β*-tubulin (labelled with KK114, in red) and the nuclear pore complex (labelled with ATTO 647 N, in green) in a mammalian cell. FLIM-based separation of fluorophores enables 2-color STED without the need for a second set of excitation-depletion lasers. The fluorescence lifetime of ATTO 647 N is shorter by about 0.8 ns compared to KK114. Scale bars: 3 *μ*m. Adapted from Gorlitz *et al* [124].

FLIM can also benefit from, or add valuable information to, other imaging modalities: for example, the combination of FLIM with super-resolution techniques like STimulated Emission Depletion (STED) allows for the characterization of the fluorophore micro-environment with a spatial resolution surpassing the diffraction limit [123]. Here, the STED principle is used to improve spatial resolution and the time-resolved signal collection permits the fluorescence lifetimes to be estimated from the signal. This can be useful for the biophysical mapping of small structures or for the visualization of FRET interactions over small spatial domains. Interesting examples of how time-resolved FLIM information can be used to enhance STED imaging capability were shown recently [124–126]. Here, the lifetime provides a way to multiplex STED measurements and to discern multiple labels: performing STED in two- or three-colors requires additional excitation-depletion lasers, adding impractical complexity for most applications. FLIM provides an elegant solution to circumvent this problem, permitting two, or even three, fluorophores with similar excitation-emission profiles to be discriminated if their fluorescence lifetimes differ. The unmixing of labels is performed computationally post-acquisition [127]. Good signal-to-noise ratio is an important requirement for this approach to be successful as the low number of photons collected per pixel in STED can be a limiting factor in the accurate estimation of lifetimes. As an example, the method has been demonstrated [124] (see figure 7) for samples containing ATTO647N and KK114 dyes, which both use the same excitation-depletion lasers but feature distinct lifetimes that can be easily distinguished with a TCSPC-STED or gated-STED microscope.

An alternative way to achieve super-resolution FLIM was demonstrated in a recent publication [28] using a 5 × 5 SPAD array to merge FLIM with Image Scanning Microscopy (ISM) [128]. The use of multi-channel timing electronics and combining the readout from multiple SPADs provided super-resolved FLIM images and a higher signal-to-noise ratio compared to confocal-based TCSPC. As SPAD arrays become commercialised, super-resolution FLIM can become accessible without requiring complex instrumentation. Pseudo-superresolved FLIM images [129] have also been reported in the literature by merging the diffraction-limited FLIM images with super-resolved intensity images acquired using Structured Illumination Microscopy.

In addition to enhancing FLIM images with super-resolved spatial information, FLIM can also be combined with other microscopy modalities providing an enhancement in image contrast. Light sheet microscopy [44, 45], LSM, has been combined with FLIM before, where the light sheet provides good intensity contrast through optical sectioning, as well as excellent acqusition speed and low phototoxicity. FLIM was used to provide concentration-independent functional contrast for *in vivo* imaging [130]. The fast acquisition speed of light-sheet microscopy makes it most suitable for combination with fast time-gated [64] or FD-FLIM approaches [63, 130].

The image contrast for FLIM can also be improved in combination with total internal reflection fluorescence microscopy (TIRFM) using high numerical aperture objectives or prisms [131]. One of the main limitations of TIRFM is the small field of view but in a recent development, the use of waveguide chips has enabled large field of view TIRFM [132, 133]. In future, such chips could serve as a powerful platform for FLIM imaging of hundreds of cells at a time with high contrast. The contrast enhancement may prove particularly useful in the investigation of membrane-bound proteins or receptor based FRET studies.

In studies involving biomechanics and characterisation of biomaterials, the combination of FLIM with Atomic Force Microscopy (AFM) provides complementary information difficult to achieve with either technique individually. AFM is a label-free technique and therefore non-specific, but provides spatial resolution *(x, y, z)* on the nanometer scale as well as information on the mechanical properties of samples. In contrast, FLIM as a fluorescence technique can provide molecular specificity but only with diffraction-limited resolution. FLIM can also assist in finding regions of interest through the fluorescence readout before scanning the sample with an AFM. The concept of correlative AFM-FLIM was first proposed in 1995 [134], and was mostly explored in the context of tip-enhanced near-field FLIM [135]. The technique found some early applications [136, 137] but with limited results from both AFM and FLIM microscopies. However, there are promising recent publications using correlative AFM-FLIM for measuring mechanical properties of oesophageal cells [138] and for assessing the topography and fluorescence quenching of chlorophyll-protein antenna complexes [139].

**Figure 8.**
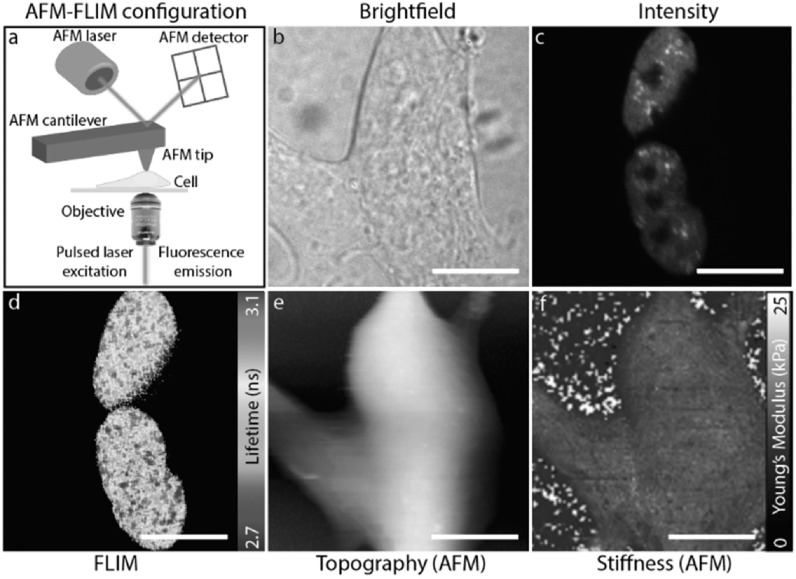
Correlative AFM and FLIM imaging of neuron like SHSY-5Y cells expressing the intranuclear protein FUS labelled with YFP. Aberrations in the phase transitioning behaviour of FUS is associated with neurodegenerative disease such as amyotrophic lateral sclerosis, ALS. Panel (a) shows a schematic of the AFM-FLIM setup on an inverted microscope. (b) Bright field image of the cell outlines. (c) Fluorescence image of FUS-YFP, demonstrating the localisation of the protein to the nucleus. (d) FLIM image of FUS-YFP which informs on the phase state of the protein. (e) and (f) Show images obtained by AFM informing on cell topology and cell stiffness, respectively. Together, the mechanical and lifetime data inform on relationships between cellular phenotypes and phase transitioning behaviour of FUS. Scale bar: 10 *μ*m.

An example from our own work is highlighted in figure 8 where this correlative technology is used to investigate nuclear biomechanics when certain proteins undergo phase separation from soluble to hydrogel to large fibrillar structures in the nucleus. Performing FLIM and AFM measurements in the same field-of-view provides a powerful means of characterising and correlating cellular mechanics with nuclear functions. Recent advances in AFM technology have allowed for smoother integration with inverted optical microscopes [140], and more accurate imaging of biological specimens, including living cells. The challenge, as in any correlative microscopy using multiple modalities, lies in registering the images acquired from the two microscopes. This is not a straighforward task because the two images can differ greatly in appearance, image content, pixel resolution, orientation, field of view, etc [141]. Semi-automated rigid and non-rigid transformation algorithms for registering multimodal data using specific cell features have been developed, especially for the broader field of correlative light and electron microscopy [142]. These tools can be adapted for correlative AFM-FLIM applications as well.

## 4. Conclusions and outlook

The observation of dynamic biological processes at subcellular resolution and the increasing throughput requirements in modern biochemical screening create a need for novel FLIM technologies that acquire data rapidly and that can be integrated with other imaging modalities. Electronic dead times and photon pileup are the primary reasons for limited acquisition speed in classical single photon counting FLIM systems. In this article, a number of recent and emerging technologies are discussed that address these problems. The field is driven by advancements made both in the underpinning hardware technologies that permit a parallelisation of the FLIM acquisition process as well as concomitant data processing and visualisation technologies. Modern implementations allow for increasing the photon count rates at which images can be acquired and combinations with other modalities such as light-sheet imaging are beginning to make an impact in the study of live cell dynamics, offering capabilities unthinkable only a few years back. Further improvements in speed, dynamic range and spatial resolution are possible as technology matures.

Whilst speed is of primary importance both in fundamental life science research and in the clinic, an ability to measure multiple biochemical parameters at once is an equally important technology driver. Correlative methods that measure intensity, spectrum, lifetime, polarisation state, etc offer formidable opportunities in modern screening applications. In these applications the low throughput speed of FLIM has often been the major bottleneck. Better timing circuitry, novel detectors such as hybrid PMTs and the parallelisation of detector and timer units, for example in SPAD arrays, offer great opportunities here. Some of these technologies are still in active development and their full potential has not been reached. A purpose of this review has therefore been to provide the reader with a comparison of the limitations as well as the advantages of the individual technologies so that the best possible methodology be adopted for a given problem.

With the advancements in FLIM throughput rates and sensitivity, there is particular potential now to combine the method with super-resolution modalities such as STED, or with endoscopic delivery for clinical diagnostics. Correlative methods that combine non-optical modalities with FLIM are also on the horizon, e.g. to combine force mapping by AFM with FLIM to relate cellular phenotypes to underlying molecular function.

These are exciting times for FLIM and despite decades of active developments in the field there is a current surge which is driven by advances in underpinning optical technologies and improved analysis methods. Much of this is still going on in specialised research laboratories and instruments require specialist expertise for operation. However, manufactures are quick to adopt the new modalities and the capabilities of commercial systems are rapidly improving. We can expect that in future a capability for FLIM is included as standard in commercial fluorescence microscopy systems.
